# Rumble: Prevalence and Correlates of Group Fighting among Adolescents in the United States

**DOI:** 10.3390/bs5020214

**Published:** 2015-05-04

**Authors:** Matt DeLisi, Michael G. Vaughn, Christopher P. Salas-Wright

**Affiliations:** 1 Department of Sociology, Iowa State University, 203A East Hall, Ames, IA 50011, USA; 2 School of Social Work, College for Public Health and Social Justice, Saint Louis University, Tegeler Hall 316, St. Louis, MO 63103, USA; E-Mail: mvaughn9@slu.edu; 3 School of Social Work, The University of Texas at Austin, 1925 San Jacinto Blvd., Austin, TX 78712, USA; E-Mail: salaswright@utexas.edu

**Keywords:** fighting, assault, group violence, criminal careers, externalizing disorders

## Abstract

Objective. Group fighting is portrayed as a piece of Americana among delinquent youth, but the behavior produces significant multifaceted negative consequences. The current study examines the heterogeneity and correlates of group fighting using national-level data. Method. Employing data from the National Survey on Drug Use and Health between 2002 and 2013 (*n* = 216,852), we examine links between group fighting and temperamental, parental, and academic factors as well as other externalizing behaviors (*i.e.*, violence, crime, substance use). Results. The prevalence of group fighting in the United States is 14.8% with 11.33% reporting 1–2 group fights and 3.46% reporting 3+ group fights. A clear severity gradient in school functioning and academic performance, sensation seeking, parental disengagement, violence and delinquency, and substance use disorders is seen in the normative, episodic, and repeat offender groups. Conclusions. Youths who participate in 3+ group fights display the exceptionality and severity of other serious/chronic/habitual antisocial youth which suggests that group fighting should be considered a significant indicator of developing criminality.

## 1. Introduction

Popularized in films such as *West Side Story, The Warriors*, *Rumble Fish*, and The *Outsiders*, group fighting or participation in a “rumble” has been portrayed as a relatively normative part of adolescence particularly among youth who are associated with delinquent gangs. Group fighting is defined as interpersonal assault with multiple participants often but not limited to a gang context and/or use of weapons. Group fighting or participating in a rumble has also appeared in delinquency research especially during the middle decades of the twentieth century [1,2,3,4,5] often as a risk factor for gang involvement and general deviance during adolescence.

In an influential conceptual taxonomy of variants of externalizing behavior [6,7,8], participating in a rumble would not generally be part of the behavioral repertoire of normative deviance displayed by adolescence-limited offenders. Normative delinquency involves flirtations with emerging adult status and is generally limited to status offenses, traffic violations, alcohol use, marijuana use, and minor nuisance offending, such as vandalism. Instead, group fighting would more likely be part of the delinquent career of a life-course-persistent offender and would be completely missing from the behavioral patterns of abstainers who have zero or marginal involvement in externalizing conduct [9,10,11].

Recent empirical research supports these theoretical ideas. Using a sample of ~20,000 participants from the National Survey on Drug Use and Health (NSDUH), Vaughn, Salas-Wright, DeLisi, and Maynard [12] conducted latent class analyses and reported four classes of delinquents. This was a normative group with limited externalizing behaviors comprising nearly 73% of the sample, a substance-using group comprising 13% of the sample, a violent group comprising over 9% of the sample, and a severe externalizing group comprising less than 5% of the sample. One of the sharpest behavioral differences among these latent classes was group fighting. About 5% of normative and substance-using youth at some point engaged in a group fight; however, between 80% and 90% of youth in the severe and violent groups had participated in group fights in the prior 12 months. In other words, group fighting is about 18 times more prevalent among severely antisocial youth than normative controls.

Given the short-term and long-term negative consequences of group fighting, including reduced neurocognitive functioning, intelligence declines, juvenile justice system involvement, disruption or termination of educational career due to expulsion, injury, and death [13,14,15,16] and the potential that this particular form of delinquency is a marker of serious criminality, more research on this behavior is needed. Using multiple samples of national-level data and rigorous quantitative methods, the current study examined heterogeneity of group fighting and its sociodemographic and behavioral correlates.

## 2. Method

### 2.1. Sample and Procedures

Study findings are based on data from the National Survey on Drug Use and Health [17] collected from non-overlapping samples drawn yearly between 2002 and 2013. The NSDUH provides population estimates of substance use and health-related behaviors in the U.S. general population. It utilizes multistage area probability sampling methods to select a representative sample of the U.S. civilian, non-institutionalized population aged 12 years or older for participation in the study. Multistage sampling designs are commonly used when attempting to provide nationally representative estimates. This is because interviewing all participants is not feasible so larger units are the first stage selected from with subsequent levels of strata partitioned until individuals from households are selected. With respect to the NSDUH, all 50 states and the District of Columbia were employed. Study participants include household residents; residents of shelters, rooming houses, and group homes; residents of Alaska and Hawaii; and civilians residing on military bases.

NSDUH study participants were interviewed in private at their places of residence. Potential participants were assured that their names would not be recorded and that their responses would be kept strictly confidential. The NSDUH interview utilizes a computer-assisted interviewing (CAI) methodology to increase the likelihood of valid respondent reports of illicit drug use behaviors [17]. The CAI methodology includes a combination of computer-assisted personal interviewing (CAPI) and audio computer-assisted self-interviewing (ACASI) methodologies. ACASI is designed to provide the respondent with a highly private and confidential means of responding to questions and is used for questions of a sensitive nature (e.g., substance use, violence, delinquency). A total of 668,012 respondents aged 12 years or older completed the survey between 2002 and 2013. The current study restricted analyses to adolescents between the ages of 12 and 17 (*n* = 216,852).

### 2.2. Measures

#### 2.2.1. Group Fighting

Respondents were asked: “In the past 12 months, how many times have you taken part in a fight where a group of your friends thought against another group?” Respondents who reported no participation in group fights were coded as 0. Those reporting some involvement were categorized as either episodic group fighters (1–2 fights; coded as 1) or repeat offenders (3+ fights; coded as 2).

#### 2.2.2. Sensation Seeking.

Two items were used to assess sensation seeking. These items include: “How often do you get a real kick out of doing things that are a little dangerous?” and “How often do you like to test yourself by doing something a little risky?” Consistent with previous NSDUH-based studies [18] the response options for each of these items were dichotomized so as to enhance interpretability.

#### 2.2.3. Parental Disengagement

Four items were used to measure parental disengagement. Sample items include: “During the past 12 months, how often did your parents limit the amount of time you went out with friends on school nights?” and “During the past 12 months, how often did your parents tell you they were proud of you for something you had done?” Youth reporting a consistent lack of parental reinforcement/control (*i.e.*, “seldom” or “never”) were coded as 1 and all others (*i.e.*, “always” or “sometimes”) were coded as 0. Previous NSDUH-based studies of parental involvement have utilized a similar dichotomization procedure [19].

#### 2.2.4. School Disengagement

Four items were utilized to measure school disengagement. Sample items include: “How interesting do you think most of your courses at school during the past 12 months have been?” and “How important do you think the things you have learned in school during the past 12 months are going to be to you later in life?” As with previous studies, these items were also dichotomized to reflect engagement *versus* disengagement [20]. We also examined academic performance on the basis of the following question: “What were your grades for the last semester or grading period you completed?” Response options included: “A average” (0), “B average” (1), “C average” (2), and “D average or lower” (3).

#### 2.2.5. Violence and Delinquency

Four items were used to assess youth involvement in other forms of violence and delinquency. Sample items include: “During the past 12 months, how many times have you attacked someone with the intent to seriously hurt them?” and “During the past 12 months, how many times have you sold illegal drugs?” These variables were coded to reflect no involvement (no participation; coded as 0), episodic involvement (1–2 times; coded as 1) and repeated involvement (3+ times; coded as 2).

#### 2.2.6. Substance Use Disorders

We examined past 12-month measures of alcohol, cannabis, and other illicit drug use disorder based on the Diagnostic and Statistical Manual of Mental Disorders, 4th edition (DSM-IV) criteria [21]. The NSDUH measures of substance use disorders are based on a battery of questions related to core DSM diagnostic criteria (e.g., unable to cut down or stop using substance, continued to use substance, even though it was causing problems, *etc.*). Prior research suggests that these measures of substance use disorders have good validity and reliability [22,23].

#### 2.2.7. Sociodemographic Factors

Demographic variables included: age, race/ethnicity, total annual household income, and the absence of father from the household.

### 2.3. Statistical Analyses

Weighted prevalence estimates and standard errors were computed using Stata 13.1 SE software. This system implements a Taylor series linearization to adjust standard errors of estimates for complex survey sampling design effects including those found in clustered data. A series of multinomial logistic regression analyses were conducted to compare youth reporting no participation in group fighting (reference group) with episodic group fighters (1–2 fights) and repeat offenders (3+ fights) in terms of sociodemographic, psychosocial, and externalizing behavioral characteristics. Specifically, we used the “mlogit” command in Stata 13.1SE with the three-level group fighting variable specified as the dependent variable and the sociodemographic factors included as control variables. Psychosocial and externalizing behavioral variables were specified as independent variables. It should be noted that results from a sensitivity analysis conducted with the group fighting variable coded as a dichotomous construct (0 = no group fighting, 1 = one or more group fights) reflected those of the analyses conducted with thee three-level measure of group fighting. However, we concluded that dichotomizing the group fighting variable masked important psychosocial and externalizing behavioral differences between episodic and repeat offender youth. As such, in the current manuscript we elected to report only the more nuanced and informative results from the multinomial (three-level) analysis. All regression analyses were conducted while controlling for sociodemographic factors, including age, gender, race/ethnicity, household income, and the absence of a father in household. Adjusted risk ratios (RRs) and 95% confidence intervals (CIs) are presented to reflect association strength. RRs were considered statistically significant only if associated confidence intervals did not include the value 1.0.

## 3. Results

### 3.1. What are the Sociodemographic Characteristics of Group Fighters in the United States?

Table 1 displays the adjusted risk ratios of adolescents reporting episodic and repeated group fighting over the previous 12 months. The prevalence of group fighting was 14.80% with 11.33% reporting 1–2 group fights and 3.46% reporting three or more group fights. Compared to the reference group (*i.e.*, youth reporting no group fights), episodic group fighters were less likely to be late adolescents (RR = 0.93, 95% CI = 0.90–0.97) and significantly more likely to be male (RR = 1.18, 95% CI = 1.14–1.23), African American (RR = 1.18, 95% CI = 1.12–1.24) or Hispanic (RR = 1.11, 95% CI = 1.06–1.17), reside in households earning less than $75,000 per year, and report no father in household (RR = 1.09, 95% CI = 1.04–1.14). Repeat offenders were also significantly more likely to be male (RR = 1.67, 95% CI = 1.57 to 1.78), African-American (RR = 1.57, 95% CI = 1.45–1.71) or Hispanic (RR = 1.32, 95% CI = 1.21–1.44), reside in households earning less than $75,000 per year, and report no father in household (RR = 1.17, 95% CI = 1.09–1.25).

### 3.2. Psychosocial Risk Correlates of Group Fighting among Adolescents in United States

Table 2 displays the adjusted risk ratios for adolescent group fighters in terms of sensation seeking and parental disengagement. With respect to sensation seeking, significant differences were observed for both episodic and repeat offender youth compared with the reference group (*i.e.*, youth reporting no group fights). Notably, the risk ratios for repeat offenders were markedly greater than those of episodic group fighters. Supplementary analyses (not shown) revealed that repeat offenders were significantly more likely than episodic fighters report frequent enjoyment of dangerous things (RR = 1.51, 95% CI = 1.40–1.62) and testing oneself by doing risky things (RR = 1.51, 95% CI = 1.40–1.62).

Group fighting adolescents were also significantly more likely to report experiencing overall parental disengagement. However, evidence suggests a stronger link between group fighting and parental disengagement among repeat offender youth than among episodic group fighters. Specifically, the adjusted risk ratios for episodic group fighters ranged from 1.18 to 1.58 whereas the risk ratios for repeat offenders range from 1.69 to 2.37. Supplementary analyses (not shown) contrasting these two groups while controlling for social demographic factors also revealed significant differences between episodic group fighters and repeat offender youth for all parental disengagement measures with odds ratios ranging from 1.43 (parents limit time out) to 1.63 (parents express pride).

Table 3 displays the associations between group fighting and measures of school disengagement and academic performance. Episodic group fighters and repeat offender youth were significantly more likely to report school disengagement and lower academic performance. With respect to school disengagement, the adjusted risk ratios for episodic group fighters range from 1.61 to 1.80 while the adjusted risk ratios for repeat offender youth range from 2.58 to 3.00. Supplementary analyses (not shown) revealed that, compared to episodic group fighters, repeat offender youth were significantly more likely to report school disengagement with adjusted risk ratios ranging from 1.53 (schoolwork meaningful/important) to 1.66 (felt overall about school). In terms of academic performance, it is noteworthy that—compared to youth reporting no group fights—episodic group fighters (RR = 3.30, 95% CI = 3.05–3.57) and repeat offender youth (RR = 6.46, 95% CI = 5.70–7.32) were significantly more likely to have a “D” average or lower.

Table 4 displays the associations between group fighting and measures of violent and delinquent behavior. Controlling for age, gender, race/ethnicity, household income, father in household, and other violent and delinquent behaviors, episodic and repeat offender youth were significantly more likely to report having attacked to seriously harm, carried a handgun, sold illegal drugs, and stolen something worth $50 or more. Among repeat offender youth, particularly large adjusted risk ratios were observed for attacking to seriously harm (1–2 times: RR = 6.92, 95% CI = 6.34–7.55; 3+ times: RR = 20.70, 95% CI = 17.94–23.88) and carrying a handgun (1–2 times: RR = 3.34, 95% CI = 2.90–3.84; 3+ times: RR = 5.09, 95% CI = 4.31–6.00).

**Table 1:** Sociodemographic characteristics of episodic and repeated group fighting adolescents in the United States.

	**How Many Times Have You Taken Part in a Fight Where a Group of Your Friends Fought against Another Group?**									
	**No Group Fights** (0 Fights)		**Episodic** *(1–2 Fights)*				**Repeat Offender** *(3+ Fights)*			
	(*n* = 182,868; 85.20%)		(*n* = 25,056; 11.33%)				(*n* = 7923; 3.47%)			
	%	95% CI	%	95% CI	RR	(95% CI)	%	95% CI	RR	(95% CI)
**Sociodemographic Factors**										
*Age*										
12–14 years	48.93	(48.62–49.23)	50.76	(49.92–51.59)	1.00		47.73	(46.24–49.22)	1.00	
15–17 years	51.07	(50.77–51.38)	49.24	(48.41–50.08)	**0.93**	**(0.90–0.97)**	52.27	(50.78–53.76)	1.06	(0.99–1.12)
*Gender*										
Female	49.84	(49.54–50.15)	45.82	(44.99–46.65)	1.00		37.60	(36.18–39.04)	1.00	
Male	50.16	(49.85–50.46)	54.18	(53.35–55.01)	**1.18**	**(1.14–1.23)**	62.40	(60.96–63.82)	**1.67**	**(1.57–1.78)**
*Race/Ethnicity*										
Non-Hispanic white	60.06	(59.75–60.36)	54.90	(54.06–55.74)	1.00		47.14	(45.67–48.62)	1.00	
African-American	14.05	(13.84–14.26)	17.58	(16.97–18.22)	**1.18**	**(1.12–1.24)**	23.21	(22.00–24.47)	**1.57**	**(1.45–1.71)**
Hispanic	18.46	(18.20–18.72)	21.03	(20.30–21.78)	**1.11**	**(1.06–1.17)**	23.84	(22.47–25.28)	**1.32**	**(1.21–1.44)**
Other	7.43	(7.26–7.61)	6.49	(6.04–6.97)	**0.91**	**(0.84–0.99)**	5.80	(5.16–6.51)	0.91	(0.80–1.03)
*Household Income*										
<$20,000	16.37	(16.14–16.60)	21.36	(20.68–22.05)	**1.45**	**(1.36–1.54)**	27.45	(26.12–28.82)	**2.21**	**(2.00–2.47)**
$20,000–$49,999	31.51	(31.23–31.80)	35.03	(34.24–35.83)	**1.29**	**(1.23–1.36)**	37.81	(36.38–39.27)	**1.76**	**(1.61–1.93)**
$50,000–$74,999	18.50	(18.27–18.74)	16.38	(15.79–16.99)	**1.07**	**(1.01–1.13)**	14.89	(13.90–15.93)	**1.29**	**(1.17–1.43)**
>$75,000	33.61	(33.32–33.90)	27.23	(26.49–27.99)	1.00		19.85	(18.69–21.07)	1.00	
*No Father in Household*										
No	74.81	(74.55–75.08)	69.89	(69.13–70.64)	1.00		64.16	(62.74–65.56)	1.00	
Yes	25.19	(24.92–25.45)	30.11	(29.36–30.87)	**1.09**	**(1.04–1.14)**	35.84	(34.44–37.26)	**1.17**	**(1.09–1.25)**

Note: Youth reporting no group fights specified as base category for multinomial regression. Risk ratios (RR) adjusted for adjusted for age, gender, race/ethnicity, household income, and father in household. RR and 95% confidence intervals (95% CI) in bold are statistically significant.

**Table 2:** Comparisons of sensation seeking and parental factors for episodic and repeated group fighting adolescents in the United States.

	**How Many Times Have You Taken Part in a Fight Where a Group of Your Friends Fought against Another Group?**									
	**No Group Fights** (0 Fights)	**Episodic** *(1–2 Fights)*					**Repeat Offender** *(3+ Fights)*			
	(*n* = 182,868; 85.20%)	(*n* = 25,056; 11.33%)					(*n* = 7923; 3.47%)			
	%	95% CI	%	95% CI	RR	(95% CI)	%	95% CI	RR	(95% CI)
**Sensation Seeking**										
*Get a real kick out of doing dangerous things*										
Never /Seldom	65.05	(64.76–65.34)	46.32	(45.49–47.16)	1.00		37.22	(35.79–38.68)	1.00	
Sometimes/always	34.95	(34.66–35.24)	53.68	(52.84–54.51)	**2.33**	**(2.24–2.42)**	62.78	(61.32–64.21)	**3.51**	**(3.29–3.75)**
*Like to test yourself by doing risky things*										
Never/Seldom	70.52	(70.24–70.80)	51.04	(50.21–51.88)	1.00		41.23	(39.77–42.71)	1.00	
Sometimes/always	29.48	(29.20–29.76)	48.96	(48.12–49.79)	**2.41**	**(2.33–2.51)**	58.77	(57.29–60.23)	**3.64**	**(3.41–3.88)**
**Parental Disengagement**										
*Parents check if you’ve done your homework*										
Sometimes/always	80.62	(80.37–8.87)	76.68	(75.94–77.39)	1.00		70.26	(68.82–71.66)	1.00	
Seldom/never	19.38	(19.13–19.63)	23.32	(22.61–24.06)	**1.33**	**(1.27–1.39)**	29.74	(28.34–31.18)	**1.92**	**(1.78–2.06)**
*Parents limit time out with friends on school nights*										
Sometimes/always	70.68	(70.39–70.97)	67.16	(66.34–67.97)	1.00		58.60	(57.04–60.15)	1.00	
Seldom/never	29.32	(29.03–29.61)	32.84	(32.03–33.66)	**1.18**	**(1.13–1.23)**	41.40	(39.85–42.96)	**1.69**	**(1.58–1.81)**
*Parents let you know when you’ve done a good job*										
Sometimes/always	86.98	(86.77–87.18)	81.00	(80.34–81.64)	1.00		73.84	(72.53–75.12)	1.00	
Seldom/never	13.02	(12.82–13.23)	19.00	(18.36–19.66)	**1.58**	**(1.51–1.65)**	26.16	(24.88–27.47)	**2.37**	**(2.20–2.54)**
*Parents say they are proud of you for something you’ve done*										
Sometimes/always	86.48	(86.26–86.69)	81.16	(80.50–81.80)	1.00		72.67	(71.32–73.97)	1.00	
Seldom/never	13.52	(13.31–13.74)	18.84	(18.20–19.50)	**1.50**	**(1.43–1.57)**	27.33	(26.03–28.68)	**2.43**	**(2.26–2.61)**

**Note:** Youth reporting no group fights specified as base category for multinomial regression. Risk ratios (RR) adjusted for adjusted for age, gender, race/ethnicity, household income, and father in household. RR and 95% confidence intervals (95% CI) in bold are statistically significant.

**Table 3:** Comparisons of school disengagement and academic outcomes for episodic and repeated group fighting adolescents in the United States.

	**How Many Times Have You Taken Part in a Fight Where a Group of Your Friends Fought against Another Group?**									
	**No Group Fights** (0 Fights)		**Episodic** *(1–2 Fights)*				**Repeat Offender** *(3+ Fights)*			
	(*n* = 182,868; 85.20%)		(*n* = 25,056; 11.33%)				(*n* = 7923; 3.47%)			
	%	95% CI	%	95% CI	RR	(95% CI)	%	95% CI	RR	(95% CI)
**School Disengagement**										
*Felt overall about going to school*										
Kind of liked/liked a lot	82.47	(82.23–82.71)	72.77	(72.00–73.53)	1.00		62.46	(60.93–63.96)	1.00	
Hated/didn’t like very much	17.53	(17.29–17.77)	27.23	(26.47–28.00)	**1.80**	**(1.73–1.88)**	37.54	(36.04–39.07)	**3.00**	**(2.80–3.21)**
*Felt that the school work was meaningful and important*										
Sometimes/always	80.92	(80.67–81.17)	72.50	(71.73–73.26)	1.00		64.29	(62.80–65.76)	1.00	
Never/seldom	19.08	(18.83–19.33)	27.50	(26.74–28.27)	**1.68**	**(1.61–1.75)**	35.71	(34.24–37.20)	**2.57**	**(2.40–2.75)**
*Importance of things learned in school for later life*										
Somewhat/very important	89.09	(88.89–89.28)	84.38	(83.74–84.99)	1.00		78.20	(76.89–79.45)	1.00	
Very/somewhat unimportant	10.91	(10.72–11.11)	15.62	(15.01–16.26)	**1.61**	**(1.53–1.70)**	21.80	(20.55–23.11)	**2.58**	**(2.38–2.80)**
*How interesting were courses at school*										
Somewhat/very interesting	78.88	(78.63–79.14)	69.62	(68.82–70.41)	1.00		61.02	(59.49–60.54)	1.00	
Very/somewhat boring	21.12	(20.86–21.37)	30.38	(29.59–31.18)	**1.70**	**(1.64–1.77)**	38.98	(37.46–40.51)	**2.64**	**(2.47–2.82)**
**Academic Performance**										
*Grades for last semester/grading period*										
An “A” average	32.62	(32.31–32.92)	19.09	(18.40–19.80)	1.00		13.60	(12.57–14.70)	1.00	
A “B” average	42.71	(42.39–43.03)	40.51	(39.64–41.38)	**1.57**	**(1.49–1.65)**	33.97	(32.45–35.53)	**1.67**	**(1.50–1.85)**
A “C” average	19.76	(19.50–20.01)	30.22	(29.40–31.04)	**2.46**	**(2.32–2.61)**	35.16	(33.62–36.73)	**3.35**	**(3.00–3.73)**
A “D” average or lower	4.92	(4.78–5.06)	10.18	(9.66–10.74)	**3.30**	**(3.05–3.57)**	17.27	(16.09–18.52)	**6.46**	**(5.70–7.32)**

**Note:** Youth reporting no group fights specified as base category for multinomial regression. Risk ratios (RR) adjusted for adjusted for age, gender, race/ethnicity, household income, and father in household. RR and 95% confidence intervals (95% CI) in bold are statistically significant.

**Table 4:** Comparisons of violent and delinquent behavior for episodic and repeated group fighting adolescents in the United States.

	***How Many Times Have You Taken Part in a Fight Where a Group of Your Friends Fought against Another Group?***									
	**No Group Fights** (0 Fights)		**Episodic** *(1–2 Fights)*				**Repeat Offender** *(3+ Fights)*			
	(*n* = 182,868; 85.20%)		(*n* = 25,056; 11.33%)				(*n* = 7923; 3.47%)			
	%	95% CI	%	95% CI	RR	(95% CI)	%	95% CI	RR	(95% CI)
**Violence and Delinquency**										
*Attack to seriously harm*										
0 times	95.84	(95.71–95.96)	80.91	(80.25–81.56)	1.00		57.87	(56.39–59.34)	1.00	
1–2 times	3.65	(3.54–3.77)	16.62	(16.00–17.25)	**4.06**	**(3.82–4.30)**	24.50	(23.26–25.79)	**6.92**	**(6.34–7.55)**
3+ times	0.51	(0.47–0.56)	2.47	(2.23–2.73)	**3.43**	**(2.97–3.96)**	17.63	(16.50–18.81)	**20.70**	**(17.94–23.88)**
*Carry a handgun*										
0 times	97.91	(97.82–97.99)	92.60	(92.14–93.03)	1.00		78.66	(77.38–79.89)	1.00	
1–2 times	1.43	(1.36–1.50)	5.48	(5.10–5.89)	**2.29**	**(2.07–2.54)**	10.69	(9.78–11.67)	**3.34**	**(2.90–3.84)**
3+ times	0.66	(0.62–0.71)	1.92	(1.71–2.15)	**1.86**	**(1.60–2.16)**	10.65	(9.73–11.65)	**5.09**	**(4.31–6.00)**
*Sell illegal drugs*										
0 times	98.10	(98.02–98.18)	91.39	(90.92–91.85)	1.00		80.48	(79.24–81.66)	1.00	
1–2 times	1.06	(1.00–1.12)	5.02	(4.67–5.39)	**2.93**	**(2.64–3.27)**	6.39	(5.70–7.16)	**2.43**	**(2.03–2.92)**
3+ times	0.84	(0.79–0.90)	3.59	(3.29–3.91)	**2.35**	**(2.06–2.67)**	13.13	12.12–14.21)	**3.40**	**(2.88–4.02)**
*Stolen something ($50 or more)*										
0 times	97.36	(97.26–97.45)	89.43	(88.90–89.94)	1.00		76.96	(75.63–78.24)	1.00	
1–2 times	1.98	(1.90–2.07)	7.88	(7.44–8.35)	**2.47**	**(2.27–2.69)**	11.17	(10.24–12.17)	**2.58**	**(2.24–2.96)**
3+ times	0.66	(0.61–0.71)	2.69	(2.44–2.97)	**1.85**	**(1.58–2.16)**	11.87	(10.88–12.93)	**3.13**	**(2.59–3.79)**

**Note:** Youth reporting no group fights specified as base category for multinomial regression. Risk ratios (RR) adjusted for adjusted for age, gender, race/ethnicity, household income, father in household, and violent and delinquent behavior. RR and 95% confidence intervals (95% CI) in bold are statistically significant.

**Figure 1:**
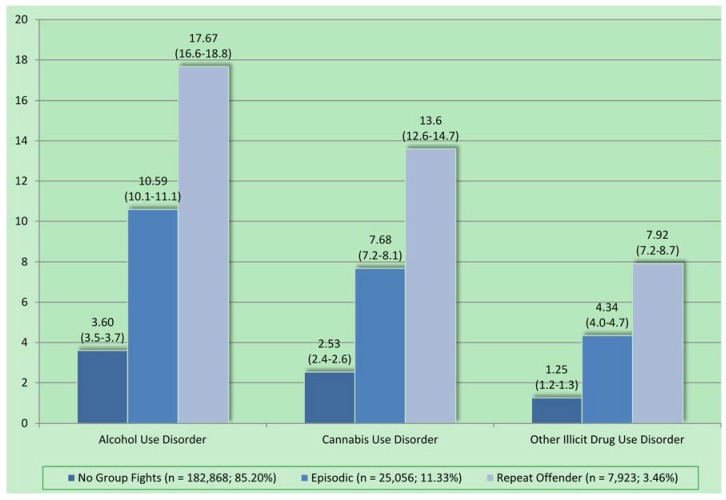
Prevalence of substance use disorders among adolescents, by level of involvement in group fighting.

Figure 1 presents the prevalence estimates for substance use disorders among adolescents reporting no group fighting, as well as episodic and repeated group fighting. Supplementary analyses (not shown) revealed that, controlling for sociodemographic factors, adolescents reporting episodic group fighting were significantly more likely than those reporting no group fights to meet criteria for alcohol (RR = 3.53, 95% CI = 3.31–3.76), cannabis (RR = 3.33, 95% CI = 3.09–3.60), and other illicit drug use disorders (RR = 3.73, 95% CI = 3.38–4.12). Compared to youth reporting no group fights, repeat offender youth were also significantly more likely to meet criteria for alcohol (AOR = 6.88, 95% CI = 6.28–7.52), cannabis (RR = 6.17, 95% CI = 5.57–6.83), and other illicit drug use disorders (RR = 7.34, 95% CI = 6.48–8.32). Comparing episodic and repeat offender youth also revealed that repeat offenders were significantly more likely to meet criteria for alcohol (RR = 1.95, 95% CI = 1.76–2.15), cannabis (RR = 1.85, 95% CI = 1.65–2.08), and other illicit drug use (RR = 1.97, 95% CI = 1.71–2.26) disorders.

## 4. Discussion

In recent years, criminology has been enriched by the advent of research using large-scale epidemiological samples such as the National Epidemiologic Survey on Alcohol and Related Conditions and the current National Survey on Drug Use and Health. Although designed to measure substance use, health-related behaviors, psychiatric problems, and personality disorders, epidemiological surveys also contain a treasure trove of measures of externalizing symptoms, conduct problems, delinquency, and criminal behaviors. Using National Survey on Drug Use and Health data between 2002 and 2013, the current study examined the prevalence and correlates of group fighting among ~200,000 participants. A series of multinomial logistic regression analyses were conducted to compare youth reporting no participation in group fighting (reference group) with episodic group fighters (1–2 fights) and repeat offenders (3+ fights) in terms of sociodemographic, psychosocial, and externalizing behavioral characteristics. All regression analyses were conducted while controlling for sociodemographic factors, including age, gender, race/ethnicity, household income, and the absence of a father in household. Adjusted risk ratios (RRs) and 95% confidence intervals (CIs) are presented to reflect association strength.

The prevalence of group fighting in the United States was 14.8% with 11.33% reporting 1–2 group fights and 3.46% reporting 3+ group fights. The normative reference group who did not participate in group fights comprised 85.2% of the sample. Episodic group fighters comprised 11.3% of the sample and repeat offenders or those who engaged in three or more group fights comprised just 3.46% of the sample. From normative to episodic to repeat offender, a clear severity gradient in school functioning and academic performance, sensation seeking, parental disengagement, violence and delinquency, and substance use disorders was seen. The normative group was characterized by higher socioeconomic status, intact family structure, gender parity, lower sensation seeking, parental engagement, school engagement, higher academic performance, and much lower prevalence of delinquency and substance use.

Episodic group fighters presented with a riskier profile evidenced by lower socioeconomic status, higher sensation seeking, greater parental disengagement, more school disengagement, lower academic performance, and more and versatile delinquency. The repeat offender group fighters presented with a substantially more severe risk profile and behavioral repertoire. These youth were disproportionately male, disproportionately African American or Hispanic, and were less likely to have a father in the household. There school disengagement was consistent and high and their academic performance was low characterized by grades of D or lower. They were significantly more sensation seeking, had greater parental disengagement, and were much more delinquent. These youth were greater than 20 times more likely to attack someone on three or more occasions, were more than five times likely to frequently carry a gun, and often sold drugs, stole, sold drugs, and used various drugs. In terms of their statistical rarity and the breadth of their antisociality, the repeat group fighters were fully consistent with prior research that similarly indicates a small cadre of extremely antisocial persons in the population [12,24,25] often referred to as career criminals, life-course-persistent offenders, or the severe 5% [6,26].

The severity gradient that is also seen in substance use disorders is consistent with the versatility that is observed in the offending patterns of serious delinquents. The prevalence of alcohol use disorder, cannabis use disorder, and other illicit drug use disorders are very low (~1%–4%) among those who do not group fight, moderate in prevalence for episodic group fighters (~4%–11%), and highest among repeat offenders (~8%–18%). This indicates that youth are not specialized in violent offending but also engage in drug use, drug selling, theft, and carrying a handgun.

Despite the strengths of the large sample (*n* = 216,852) of youth ages 12 to 17 years, there are important limitations that should be considered to contextualize the findings. For instance, extensive research in criminal justice and allied fields [7,27,28,29,30,31,32,33] has documented the diverse developmental pathways that are associated with severe externalizing behaviors such as those exhibited by the repeat offender group in the current study. We were not able to examine the antecedent conditions that manifest in repeated group fighting, and this is an important area for future investigators to consider. In addition, despite the use of Moffitt’s developmental taxonomy as a conceptual framework, the current study was not guided by a specific theory that could be used to overlay behaviors of the groups. For instance, the role of sensation seeking, disengagement from parents and school, and versatile delinquent behaviors is consistent with the theoretical ideas of low self-control [34] and low temperamental effortful control and negative emotionality [35]. Our study is also limited by the fact that the NSDUH includes only a handful of violent and delinquent behaviors. As such, we were not able to assess the association between group fighting and the full array of behaviors that delinquents might be involved in. Future studies could also model the association between theoretically-driven constructs and the various groupings in these data.

## 5. Conclusions

The current study indicates that participating in a rumble is not the violent albeit trivial event that has historically been portrayed in films, but instead is a potential marker of significant antisociality and broadband involvement in externalizing and delinquent behaviors. Indeed, more than 85% of youth never engage in such behavior. Among those who do, their school functioning, family dynamics, and behaviors are more negative and indicate multiple deficits, and among those who frequently group fight, violence is part and parcel of a highly antisocial life.
